# 

**Published:** 1886-01-20

**Authors:** 


### INDEX.

A.

Abdominal cavity, gunshot wounds of the...459
Abdominal surgery...156, 452, 459
Abdominal tumor...6
Abortion...104, 364
Abortion from sassafras...104
Abortion, prophylactic treatment of...369
Abscess, multiple...414
Abstracts and new books...75
Academy of Medicine, Nashville...280
Acid, phenic, in intermittent fever...144
Acid phosphates...16
Advertisements, our...35
Age, hygiene of old...214
Aged, insomnia of the...229
Ague...154
Alabama Medical Association...203
Albumen, tests for...146
Alcohol...17, 28, 156
Alcohol as a hypnotic...28
Alcohol weakening...156
Alcoholic stupor...17
Algin...142
Alkaloid, pomegranate...63
Alterative medication...274
Alteratives...113, 274
Amaurosis...67
Amblyopia nicotiana...107
Amenorrhœa...154, 184, 237
American Editors, Association of...116
American Medical Association...25, 156, 221
American Rhinological association...75
American women, child bearing period of the...18
Ammunition...366
Amputation...473
Anæmia...26, 237, 391
Anæmia, conception of male children in post-menstrual...391
Anæsthesia...167, 191, 337, 344
Anæsthesia, local...191
Anæsthetic, marvelous results of a new...344
Anæsthetics, administration of...167
næsthetics in labor...337
Anal circumference, excision of...341
Anatomical bill, the...141
Andrey, Dr. J. A...81, 118
Angina pectoris...188
Ani, cocoaine in pruritus...185
Aniline in epilepsy...147
Anise cordial...438
Anodyne for vesical irritation...432
Anti-epileptic and nervine, a new...16
Antipyrine...27, 105, 143, 274, 426
Antipyrine, another application for...426
Antipyrine in pregnancy...143
Antipyrine in phthisis...274
Antiseptic dressing in amputation...473
Antiseptics...219
Ants...72
Anus, painful fissure of...184
Aphthous sore mouth...74
Apone...438
Archibald, Dr. Henry C...96
Arsenic...184, 186
Arsenic habit...186
Arsenic in lymphadenoma...184
Aspiration, supra pubic...51
Association, American Medical...35, 156, 221
Association, American Rhinological...75
Association, Georgia Medical...157, 158, 198, 399
Association, National Eclectic Medical...280
Association of Alabama, Medical...203
Association of American Editors...116
Association of physicians, a new...146
Association, Stone Mountain Medical...281, 359
Association, The Texas State Medical...75, 239
Asthma...28, 263, 353, 390
Atlanta as a medical center...76
Atlanta Society of Medicine...238
Atropine...70, 144, 190
Atropine in shock...144
Atropine versus chloroform...190
Attarway, death of Dr...440
Auto-suggestion, hemorrhage by...20

B.

Baby as a water drinker...379
Bailey, Dr. William...334
Balata...157
Ballinger, death of Dr...75
Baltimore as a medical center...156
Bathers, ten commandments for...433
Baths, hot packs and pilocarpine compared...389
Battey at the Atlanta Society of Medicine, Dr...479
Bees...72, 147
Bee stings...147
Begonia...95
Belladonna in catarrh...228
Benzoate of cocoaine...188
Benzol sulphuricimide...281
Berger, Dr. J...352
Berry, Dr. J. J...176
Bi chloride of mercury in phthisis...153
Bile, that...251
Bite of a copperbelly snake...365
Bladder, gunshot wound through...1
Bladder, irritability of...154
Blind, sight for the...151
Blister quickly, how to...16
Bodies, photographing celestial...235
Bodies, removal of foreign...61, 347
Body, development of the human...435
Boracic acid in diabetes mellitus...21
Borated vaseline in erysipelas...110
Borcheim, Dr. L. Edwards...5
Bowel, a case of obstructed...326
Bradley, Dr. Benjamin A...134
Bright's Disease, exudative retinitis in...460
Bromide of potassium and calomel, incompatibility of...272
Bromine in diphtheria...230
Bronchitis, turpine in...354
Brown's iron bitters...155
Brucine...271
Bryce, Dr. C. A...379
Buboes...136, 193, 376
Buggy case, a physician's...276
Burns...251
Burt, death of Dr. W J...359

C.

Calcium chloride in glandular enlargements....190
Calcium hippurate in dyspepsia....136
Caldwell, death of Dr....117
Calomel and bromide of potassium, incompatibility of....272
Cancer, prevention of....149
Cape of Good Hope, clouds at the....476
Capitol, the Evening....280
Carbolic acid....138, 140, 393
Carbolic acid in hemorrhoids....140, 393
Carbolic acid in phthisis....138
Carbuncle....127
Carcinoma of cervix uteri....252
Caries of temporal bone....192
Carson's paint....114
Carter, Dr. C. J....207
Casanow, Dr....424
Cascara cordial....470
Cataract operation....281, 391
Cataract extraction, cocoaine in....391
Catarrh....54, 73, 155, 228, 328, 438
Catarrh, belladonna in....228
Catarrh, nasal....328
Catarrhal headache....438
Caterpillar, swallowing a....68
Catgut sutures, infection by....140
Cattle bones....236
Cautery in diphtheria....99
Celestial bodies, photographing....235
Cervix uteri, fatal results from splitting the....351
Cessation of menstruation....64
Character, the force of....480
Charleston earthquake....395, 398
Chassagnac, Dr. Chas....15
Chicago doctor, a....273
Chilblains....33, 34, 113
Childbearing period, lengthening of....18
Children, Anodyne for....74
Children, Chinese method of hardening....149
Children, coca in....350
Children, constipation in....74
Children, offensive diarrhœa in....472
China, how they do it in....438
Chinese, health of the....269
Chinese medical missions....267
Chionanthus virginicus....259
Chittick, Dr. W. R....93
Chloral hydrate, external use of....29
Chloranodyne....416
Chloroform....156, 190, 196
Chloroform, atropia versus....190
Chloroform, cheap....156
Chloroform, elixir of....196
Chlorosis in young girls....237
Cholera....73, 153, 156, 277, 392
Cholera abroad....156
Cholera infantum....153, 392
Cholera mixtures....73
Cholera morbus....277
Chorea....3, 69, 229, 272
Chorea, Hyosciamine in....229
Christopher, Dr. Hiram....54, 330
Cigar for five cents, a good....355
Cimicifuga in headache....192
Circumcision, substitute for....393
Clinical lectures, Southern Med. College....439
Clinical lectures, post-graduate N. Y. Medical school....439
Clock, magnetic....198
Cobra poison....112
Coca, a palatable preparation of....76
Coca in children....350
Cocaine....21, 63, 69, 138, 155, 165, 185, 188, 193, 227, 231, 234, 281, 363, 391, 393, 439
Cocaine addiction....439
Cocaine benzoate....188
Cocaine, convenience and economy in the use of....231
Cocaine in asthma, salicylate of....363
Cocaine in cataract extraction....281, 391
Cocaine in diarrhœa from teething....138
Cocaine in dysentery....234
Cocaine in gastric lavage....227
Cocaine in gonorrhœa....63
Cocaine in inflamed nipples....69
Cocaine in labor....393
Cocaine in pruritus ani....18[illegible]
Cocaine in throat disease....165
Cocaine in the forbidden list....21
Cocaine in tonsilotomy....193
Cocaine intoxication....21
Cocaine, price of....156
Coffee in pruritus....150
Colds, glycerine in....109
Collaborators....35
College, The Southern Medical...84, 238, 279, 359, 400
Commencement exercises, Southern Medical College....84
Commission on yellow fever....77
Conception of male children at the time of post menstrual anemia....391
Conductors, machine to make them honest....396
Conium as a nerve stimulus....106
Conjunctival inflammations....354
Constipation....472
Constipation in children....74
Consumption, inoculation in....235
Contraction, hour glass....205
Convention, sanitary....155
Convulsions of children, morphine hypodermics in....388
Cooper, Dr. Hunter P....280
Copperbelly snake, bite of a....366
Coriza....107, 189
Corneal inflammation....354
Corning on local anæsthesia....191
Corpses galvanized....151
Corrections....197, 439
Correspondent, New York special....440
Corson, Dr. Hiram....348
Cortelyon, Dr. P. R....165
Cookery, Dr. Oscar J....256
Cotton root bark in epilepsy....279
Coughs....40, 357
Coulter, Dr. J. H....369
Country doctor....116, 249
Creasote a specific for erysipelas....386
Crotonol....69
Croup....25, 154, 179, 272, 419
Croup, difference between diptheria and....419
Crow, Dr. Walter A....405
Cutaneous hemorrhage....20

D.

Daily Medical Journal....156
Dambury hats....152
Dana, Dr. C. L....209
Davis, Dr. N. S....75
Dead drunk....17
Decapitated head, experiments on a....264
De La Mater, Dr. S. T....132, 367
Delivery during hypnosis....394
Dental fistulæ....192
Detroit Gynæcological Society....357
Development of the human body....435
Diabetes....21, 136, 334
Diabetes mellitus....21, 334
Diagnosis, a....330
Diarrhœa....74, 137, 138, 142, 269, 397, 472
Diarrhœa, chronic....74
Diarrhœa, infantile....137
Diarrhœa in children, offensive....472
Diarrhœa from teething....138
Difference between diphtheria and croup...419
Digestibility of various foods....273
Digestive wine....73
Diphtheria....62, 179, 397, 431, 433, 437, 474
Diphtheria and croup, difference between...419
Diphtheria, Bromine in....230
Diphtheria continually increasing in England....110

Diphtheria, galvano-cautery in ....................99
Diphtheria, vinegar in.................................... 474
Discovery, a highly important..........................359
Discussions, Medical Society..........................186
Dislocations, reduction of.............................. 70
Diuretic, water as a......................................351
Doctor, the country.................................116, 249
Dog's reasoning, a .......................................475
Dots, practical................................................2
Drainage tube in the chest for 21 months.....256
Dressing, fixed .............................................357
Dressing in amputation, antiseptic................472
Drugs by mammary glands, excretion of.....425
Duct of steno, fistula of................................192
Dulles, Dr. Chas. W.........................................44
Durham, Dr. A. F...........................................365
Dynamite, fishing with..................................276
Dysentery.....................57, 145, 234, 277, 278, 428, 447
Dysentery, chronic........................................145
Dysentery, cocaine in.................................... 234
Dystocia, a case of...........................................57

E.

Ear, removal of foreign bodies from the......347
Ear, suppuration of middle......................187, 269
Eclampsia, infantile ......................................108
Eclampsia, puerperal......................................405
Eclectic Medical Association, National........280
Editors Association of America....................116
Egg diet.......................................................... 431
Egg register.....................................................102
Egg, the wonders of an...................................236
Electric plant..................................................108
Electric shocks, resisting...................................32
Electrical foot warmers..................................396
Electricity in labor............................................70
Endometritis................................................... 233
Enlargement, prostatic.......................................70
Enuresis nocturna...........................................145
Ephemeris, Squibb's...........................................35
Epilepsy .....................................................147, 270
Epileptic, a new anti.........................................16
Epistaxis, antipyrine in....................................105
Ergot in gonorrhœa ........................................345
Erysipelas.........................................110, 386, 394
Erysipelas, borated vaseline in........................110
Erysipelas, creasote a specific in...................... 386
Erysipelas, jaborandi in....................................394
Ethoxy caffeine...............................................348
Eucalyptus in croup............................................25
Eve, death of Dr. Joseph A.................................77
Evening C pitol..................................................280
Exophthalmic goitre...........................................11
Eye, exenteration of the.....................................110
Eye ball, foreign body in................................... 129
Eye water, Thompson's.......................................155
Explosives 190 miles away..................................155

F.

Fainting.................................................................27
Fattening on water................................................71
Feeding of infants, artificial................................193
Fennel gin...........................................................438
Fever...........31, 36, 37, 77, 144, 150, 153, 194, 273, 358
Fever, cause of....................................................273
Fever, intermittent ......................................144, 358
Fever thirst, glycerine in ......................................150
Fever, typhoid.......................................................153
Fever, yellow................................31, 36, 37, 77, 194
Fingers and toes, frostbitten...................................26
Fishing with dynamite...........................................276
Fissure of the anus, painful....................................184
Fistula, turpentine in.......................................189, 192
Flashes, hot............................................................272
Flint, death of Dr. Austin........................................156
Fluorine..................................................................476
Fœtal psychology.....................................................175
Fœtus, action of quinine on the..........................109, 260
Fœtus, determination of the sex of the.......107
Fœtus, influence of mental impression on the...........................................................427
Food for infants.....................................................357
Foods, digestibility of various..............................273
Foot warmers, electrical.........................................396
Foreign bodies in the ear........................................347
Forests, our disappearing.......................................151
Fracture, Paris plaster in treatment of.......................5
Fractures.............................................................5, 100
Frostbitten fingers and toes.......................................26
Fuel problem ...........................................................152

G.

Galvanized corpses.................................................151
Galvano-cautery in diphtheria....................................99
Gangrene...................................................................101
Gaston, Dr. J. McF.1, 127, 205, 245, 280, 341, 414, 478
Gastric lavage............................................................277
Gaynor, Dr. John J. ....................................................258
Georgia Medical Association..115, 157, 158, 198, 399
Ghosts, investigating...................................................275
Glandular enlargements...............................................190
Glass through the foot, a piece of................................101
Gleet..............................................................................429
Glycerine in cold............................................................109
Glycerine in fever thirst.................................................150
Glycerole for cutaneous pruritus.....................................438
Glycosuria..........................................................................28
Goldtsnoven, Dr. E. van......................................................277
Goitre, exophthalmic.......................................................11, 29
Gonorrhœa......29, 63, 108, 155, 266, 345, 358, 371, 437
Gonorrhœa, liquid ergot in chronic...................................345
Gouty rheumatism...............................................................136
Grains and some chaff, a few...............................................249
Granular lids........................................................................148
Granulations........................................................................186
Graves' disease......................................................................11
Graves' malpractice suit.........................................................456
Guerin, death of Jules............................................................115
Gunshot wounds.................................................................1, 24
Gunshot wounds of the abdomen..........................................459
Gunshot wound through bladder...............................................1
Guthrie's telephone...............................................................195
Gynæcological Society, Detroit................................................337
Gynæcology in the Southern Med. College..279
Gynæcology, panacea of modern..............................................220

H.

Hair, what a Frenchman can do with a......276
Hair dye, walnut...................................................................195
Hair to its original color, restoring gray......109
Hamamelis in prostatic enlargement..............................70
Hamilton, death of Dr. D. B...........................................115
Hamilton, Dr. J. L.............................................................280
Hands, cleaning..................................................................112
Harper, death of Dr. J.............................................................75
Hardon, Dr. Virgil O.....................................................252, 279
Harris, death of Mrs. Dr. N. O........................................203, 242
Hay fever and coriza..........................................................109, 186
Head, experiments on a decapitated....................................264
Headache.....................................................4, 153, 192, 273, 438
Hearing of infants......................................................................152
Hearn, Dr. W. Joseph...................................................................167
Heart, punctured wound of the......................................................15
Heddens, Dr. Jos. W.....................................................................459
Helm, Dr. Scott............................................................................376
Hematuria, malarial.....................................................................278
Hemicrania....................................................................................156
Hemisphere, the Southern.............................................................436
Hemorrhage, cutaneous...................................................................20
Hemorrhage, post partum.................................................83, 394, 421
Henning, Dr. John A......................................................................259
Hemorrhage, uterine......................................................................109
Hemorrhoids.............................................74, 90, 140, 341, 375, 393
Heredity..............................................................................................97
Hernia, to reduce..............................................................................148
Herniotomy......................................................................................207
Hiccough.........................................................................................266
Hicks, Dr. Braxton............................................................................173
Hippurate of calcium in dyspepsia, diabetes, etc..................................................................136
Hobbs, Dr. A. G...........................................................................129, 328
Hospital, Ivy street..............................................................................37

Hot Flashes....272
Hour glass contraction....205
House fly, parasites of the....475
Howard, Dr. Curtis C....374
Hydrocyanic acid, insusceptibility of dogs to....71
Hydrophobia....31, 36, 66
Hygiene of old age....214
Hyosciamine in chorea....229
Hyoscine hydrobromate....98
Hypnone....102
Hypnosis, delivery during....394
Hypnotic, alcohol as a....28
Hypodermic injection of cold water in sciatica....102
Hypodermic of quinine solution....114
Hysteria....237
Hystero-catalepsy in a male patient....274

I.

Ice to the spine in obstinate vomiting....107
Impotence, functional....19, 278
Infantile diseases, diagnosis of....134
Infants, artificial feeding of....193
Infants, food for....357
Infants, hearing of....145
Ingluvin....474
Injections in intestinal obstruction, forcible.65
Inoculation in consumption....235
Inoculation for yellow fever....36, 37, 77
Insanity, some forebodings of incipient....271
Insomnia in the aged....229
Institute in New York, a Pasteur....340
Intermittent fever....144, 358
International Medical Congress....115, 157
Intestinal obstruction....65
Investigating ghosts....275
Iodine in rheumatism....114
Ivy street hospital....87

J.

Jaborandi in erysipelas....394
Jacobi to Dr. Andrey, letter of Dr....118
Jaundice....68
Jenner's versification....274
Johnson, tribute to Dr. J. M....240
Jones, Dr. Charles N. Dixon....277, 445
Journal, Daily Medical....156

K.

Keloid....103
Kind words....478
Kiss, scarlet fever in a....29
Knee, white swelling of....343
Kola....260

L.

Labor....22, 70, 83, 112, 141, 337, 348, 363
Labor, abnormal phenomena in normal....245
Labor, anæsthetics in....337
Labor, cocaine in....393
Labor, electricity in....70
Labor, induction of premature....141
Labor, statistics of 3 636 cases of....348
Labor, strychnia before....83
Labor, tedious....22
Laborating work, cleaning hands after....112
Lactation and medicaments....190
Lallerstedt, Dr. T. L....2
Lane, Dr. Levi C....456
Lanoline....147
Lavage, Gastric....277
Leather to iron, how to glue....71
Lemonade, iron....437
Lemonade, salicylic....187
Liberal offer, a....279
Lids, granular....148
Lightning, damage done by....435
Limbs, saving condemned....224
Lithium salicylate in rheumatism....150
Lobelia inflata, external use of....110
Lobelia inflata in asthma....390
Lombard, Dr. C. S....95
London hospitals, surgical peculiarities in..445
London Ophthalmic Hospital, ophthalmology in....382
Longevity....15, 436
Lunatic asylum, Milledgeville....449
Lymphadenoma, arsenic in....184

M.

Magnesium sulphate in rheumatism....233
Magnetic clock....195
Magnetism in chemical reaction, influence of....395
Malaria, how to administer quinine in....418
Malarial fever....254
Malarial hematuria....278
Malignant pustules....178
Mammary glands, excretion of drugs by....425
Manaca in rheumatism....146
Marriage, the interval between it and the first child....147
Masturbation....51
Mattison, Dr. J. B....439
Meatus urinarius, irritation of....3
Medical and surgical history of the war....197
Medical Association, American....35, 116, 156
Medical Association of Alabama....203
Medical Association of Georgia....115, 157, 158, 198, 399
Medical Association, National Eclectic....280
Medical Association, Stone Mountain...281, 359
Medical Association, Texas....239
Medical College, the Southern..238, 275, 279, 359, 400
Medical Congress, International....115, 157
Medical Journal, Daily....156
Medical men as witnesses and experts....427
Medical missions, Chinese....267
Medical society, a new....239
Medical Society, Tennessee....36, 116
Medication, alterative....274
Medication, an improved method of....362
Medicine, Nashville Academy of....289
Memoriam, in....203
Menstruation, cessation of....64
Menthol....153, 234, 466
Menthol in headache....153
Menthol in urticaria and pruritus....234
Mercury in phthisis, bi.chloride of....153
Mercury in syphilis, tannate of....424
Metric system....38
Metrorrhagia....5
Microbe....30
Microphone vibrations reproduced by....356
Microscopical discoveries, the latest....355
Migraine....25, 348
Milk, digestion of....189
Milk, drying up the....232
Milledgeville lunatic asylum....449
Minnesota, big woods of....32
Mitchell, Dr. A....219
Moffitt, Dr. F. W....178
Moon and us, the....72
Morphia hypodermics in convulsions of children....388
Morphia in infantile eclampsia....108
Morphia in scarlet wrappers....156
Mother's milk....140
Mounteagle....280
Mouth, sore....74
Mucilage, a good....73, 113

N.

Naphthaline....137
Nasal catarrh....328
Nasal hemorrhage....192
Nashville Academy of Medicine....280
National Eclectic Medical Association....280
Nebula in the Pleiades, the new....275

Nerve sheath in sciatica, puncture of..........353
Nervine, a new.......... 16
Neuralgia..........16, 234, 250
Neurological therapeutics..........209
New Jersey children sent to Pasteur..........35
New school friends, our..........238
Neyron Dr. Joseph..........280
Nickel poison..........476
Nicolson, Dr. Wm. Perrin..........6
Nipples, inflamed..........69, 274
Nose, foreign bodies in the..........61, 232
Notes, therapeutic..........176
Nurse's sore mouth..........142

O.

Obstruction, intestinal..........65
O'Dwyer's method in croup, diphtheria, etc.179
Offer, a liberal..........279
Operation, shock after..........144
Ophthalmology in the London ophthalmic hospital..........382
Opium habit..........18, 26, 105
Opium poisoning..........18
Otorrhœa..........108, 432, 468
Otorrhoea in children, acute..........468
Otorrhœa, ulcerative..........432
Ovarian disease..........51

P.

Packs, baths and pilocarpine compared......389
Pain, reflex..........433
Panacea of modern gynæcology..........200
Paraldehyde..........56, 149 181
Paraldehyde as an antidote to strychnia......149
Paraplegia from reflex causes..........367
Parasites of the house fly..........475
Parcels post, the English..........236
Paris plaster in the treatment of fracture......5
Parthenine in facial neuralgia..........234
Parturiometer..........64
Pasteur institute in New York, a..........340
Pasteur mission, the..........231
Pasteur treatment..........273
Payne, Dr. F. T..........51
Peppermint versus cocaine..........106
Pepsin, wine of..........3t
Pericardium, tapping the.......... 130
Permanganate of potassium in snake bites.352
Phenic acid in intermittent fever..........144
Phosphates, acid..........16
Phosphorous, turpentine as an antidote to..434
Photographing celestial bodies..........235
Physicians, a new association of..........146
Physician's buggy case, a..........276
Phthisis..........138, 153, 226, 274
Pichi..........67
Pilocarpine..........70, 144, 187, 271, 389
Pilocarpine, hot baths and hot packs compared..........389
Pilocarpine injections..........187
Pilocarpine in pneumonia..........144, 271
Pimple ointment..........113
Pine, syrup of white..........114
Piperine..........434
Pitting in small pox, to prevent..........155
Placenta, retained..........461
Placenta previa..........96, 143, 173
Plant, a new electric..........108
Pleurisy..........60
Pneumonia..........27, 139, 144
Pomegranate, alkaloid..........63
Populin..........28
Post partum hemorrhage..........394
Powell, Dr. Seneca D..........371
Pregnancy, antipyrine in..........143
Pregnancy, vomiting of..........34
Pressure in dislocation..........70
Prison reform..........441
Professor, an old.......... 280
Proposition to change the organization of the Georgia Medical Association..........399
Prostatic enlargement..........70
Prostatic inflammation..........143
Pruritus..........150, 185, 196, 234, 274, 438
Pruritus ani, cocaine in..........185
Pruritus, coffee in..........150
Pruritus vulvæ..........196
Psychology, fœtal..........175
Ptomaines..........430
Puerperal eclampsia..........106
Puncture of nerve sheath in sciatica..........353
Pustules, malignant..........178

Q.

Quinine, disguising the taste of..........74, 107, 267
Quinine, fatal dose of..........63
Quinine in irritable stomach..........150
Quinine in malaria..........418
Quinine inunction in malarial fever..........254
Quinine made by synthesis..........359
Quinine solution, hypodermic of..........114

R.

Rackrock..........31
Rattan scraping, a machine for..........102
Rattlesnakes..........111
Reed, death of Dr. J. A..........35
Reflex causes, paraplegia from..........367
Reflex pain..........433
Removal of foreign bodies in nose..........61
Resorcin in gonorrhœa..........155
Responsibility? where is the..........161
Retention of urine..........61, 181
Retinitis in Bright's disease, exudative..........460
Reynolds, Dr. Dudley S..........281
Rheumatism..........29, 74, 114, 136, 146, 196, 232, 249, 270, 358, 380, 429
Ring from the penis of a boy, removal of a..193
Ringworm..........188, 233
Rosick, Dr. F. H..........90
Roy, Dr. G. G..........326

S.

Salicylate of lithia in rheumatism..........150
Salicylate of soda in sick headache..........273
Salicylates, administration of..........340
Salicylic lemonade..........187
Salix nigra..........51
Salt Springs, our trip to..........360
Sanitary convention..........156
Sassafras, abortion from..........104
Savage, Dr. G. C..........279
Scabies..........249
Scarlet fever..........29, 217, 237, 397
Sciatica..........33, 102, 268, 353
Sciatica, puncture of nerve sheath in..........353
Scott, Dr. H. F..........121
Scrotum, transfixion of..........19
Sea, danger from umbrellas at..........355
Sea depths, penetration of light into..........194
Sea sickness, a new remedy for..........20
Sea, wonders of the..........111
Septicemia..........17
Sex of the fœtus, determination of..........107
Sexes, longevity of the..........436
Shock after operation, to prevent..........144
Sight for the blind..........151
Skin diseases..........28, 234
Skin diseases, asthma in..........28
Skin diseases, spray remedies in..........234
Sleeplessness..........474
Small pox..........106, 155
Smith, death of Dr. Asbel..........115
Snake bites, permanganate of potassium in 352
Society, a new medical..........239
Society, Detroit Gynæcological..........337
Sodium silicate in spinal diseases, jackets of.132
Sodium sulphate..........227
Southern Medical College..84, 238, 275, 279, 359, 400
Soul enter the body? at what moment does the rational..........258

Sozinsky, Dr. Thos. S....... 175
Sparteine....... 270
Specialist, a....... 274
Spermatorrhœa....... 51
Spinal diseases, jackets of sodium silicate in. 132
Spinal tenderness....... 26
Spray method....... 54
Squibb's ephemeris....... 35
Squills in whooping cough....... 434
Startling disclosure....... 240
Steno's duct, fistulæ of....... 192
Stewart, Professor T. Granger....... 30
Stomach, quinine in irritable....... 150
Stomatitis materna....... 142
Stone Mountain Medical Association.... 281, 359
Strychnia, paraldehyde antidote to....... 149
Strychnia before labor....... 83
Stupor, alcoholic....... 17
Succi, Signor....... 476
Suicides....... 191
Sulphur in chronic dysentery....... 145
Suppuration of middle ear....... 187
Supra-pubic aspiration....... 61
Surgery, abdominal....... 156, 452, 459
Surgery of general practice....... 44
Surgical practice in the London hospitals... 445
Synthesis, quinine made by....... 359
Syphilis....... 345, 424
Syphilis, prevention of....... 345
Syrup of white pine....... 114
System, metric....... 38
Swelling of the knee, white....... 343
Swellings....... 136

T.

Teething....... 138
Telephone, Guthrie's....... 194
Temporal bone, caries of....... 192
Tennessee Medical Society....... 36, 115
Tetanos....... 121
Texas Medical Association....... 239
Theine....... 67
Therapeutic notes....... 177
Therapeutics and neurological therapeutics. 209
Thermo-cautery in gangrene....... 101
Thermometers....... 36
Thirst, glycerine in fever....... 150
Thompson, Dr. J. W....... 11
Thompson's eye water....... 155
Throat disease, cocaine in....... 165
Thunder storms....... 356
Tobacco....... 185
Toes, frostbitten....... 26
Tolu varnish in diphtheria....... 62
Tonic and alterative....... 113
Tonic mixture....... 114
Tonsilitis....... 148, 149, 278
Tonsillotomy, cocaine in....... 193
Tooth extraction, painless....... 70
Tooth forceps blade from right bronchus, removal of....... 104
Transfixion of scrotum....... 19
Transfusion and infusion....... 465
Transactions of the Georgia Medical Association....... 201
Tsuchiakabi....... 150
Tubercular phthisis....... 226
Tuberculosis, pulmonary....... 260
Turks, the....... 261
Turpentine in croup....... 25
Turpentine in fistula....... 189
Turpentine versus phosphorus....... 434
Turpine....... 113, 354
Typhoid condition....... 196
Typhoid fever....... 137, 153, 183, 387
Typhoid fever, veratrum viride in....... 387

U.

Umbrellas at sea, danger from,....... 355
Urethane....... 135, 385
Urine, retention of....... 61, 181
Urticaria....... 234
Uterine cervix dilation during labor....... 93

V.

Vaccination, strong argument in favor of... 141
Vaccine virus....... 68
Vaccinization....... 230
Vaginal pastiles....... 140
Vaginismus....... 357
Varicocele....... 234, 381
Vaseline in erysipelas, borated....... 110
Venery, excessive....... 51
Veracity between Dr. Battey and Mr. Lawson Tait, question of....... 240
Veratrum viride in typhoid fever....... 183
Vesical irritation....... 432
Vibrations photographed and reproduced by microphone....... 356
Vinegar in diphtheria....... 474
Vomiting, ice in obstinate....... 107
Vomiting of pregnancy....... 34, 148
Vulvæ pruritus....... 196

W.

Wakely, death of Dr. James....... 439
Walnut hair dye....... 195
Warts, to remove....... 155, 380
Water as a diuretic....... 351
Water drinker, the baby as a....... 379
Water for infants....... 145, 270
Water in corneal and conjunctival inflammations....... 354
Water in sciatica, hypodermics of....... 102
Water is fattening....... 71
Water test....... 374
What was it?....... 81
White swelling of the knee....... 343
Whitlow, nitrate of silver in....... 420
Whooping cough....... 265, 434, 438
Williams, Dr. P. C....... 421
Wine, digestive....... 73
Witnesses and experts, medical men as....... 427
Wizzard oil....... 477
Wonders of the sea....... 111
Wood, Dr. N. C....... 214
Wood, Professor H. C....... 115
Wood worms, to destroy....... 276
Wool fat....... 147
Word, Dr. R. C....... 41
Wound of the heart, punctured....... 15
Wounds, gunshot....... 24, 459
Wounds of the abdomen, gunshot....... 459

Y.

Yellow fever....... 31, 36, 37, 77, 194
Yellow fever, inoculation....... 36, 37, 77
Young, Dr. John H....... 254

*RECEIPTED.*

For 1885—Drs. J. W. Hill, Thos. S. Jones, I. M. Covington, Alford & Bro., E. M. Pharr, to October; B. E. Clark, A. B. Loving, W. N. Ames, J. J. McGhee, A. H. Sellers, W. L. Bullard, M. F. Alford, W. B. Maxwell, D. C. Montgomery, C. S. Priestly, J. S. Bruce, A. R. Jones, J. F. Brooks, to October; C. J. Church, A. A. Davidson, to April; E. P. Overby, to July; J. N. Wadsworth, to July.

For 1886—M. M. Holland, to October; M. M. Coleman, to March; J. H. Young; J. B. Benton, to April; P. Taylor, B. F. Duke, J. D. Blue, J. M. Suttles, F. Courtney, C. T. Murphey, T. S. Parrham, Lockwood Allison; J. H. Burchmore, to April; A. D. Coleman, to May; V. B. Watson, T. P. Oliver, W. T. Foute

For 1887—E. A. Anderson, to July; C, W. Keller.

For 1884—K. K. Lyons.

